# Primary Follicular Lymphoma of Thyroid: A Rare Case Report with Review of the Literature 

**DOI:** 10.30699/ijp.2024.562997.2985

**Published:** 2024-10-02

**Authors:** Shruthi K P, Lincy Joseph, Jeena V Chimmen

**Affiliations:** Department of Pathology, Jubilee Mission Medical College, KUHS, Thrissur, Kerala, India

**Keywords:** Follicular lymphoma thyroid, primary thyroid lymphoma, extranodal lymphoma

## Abstract

**Background & Objective:**

Thyroid lymphomas are predominantly secondary to lymphoma at other sites, and primary follicular lymphoma of the thyroid is a very rare entity.

**Case Presentation:**

Here, we report a case of a 62-year-old female who presented with swelling in the front of her neck for one month. The clinical diagnosis was a multinodular goiter. Fine needle aspiration cytology was done and reported as nodular colloid goiter with lymphocytic thyroiditis. The system examination was unremarkable. Histopathological assessments of the right hemithyroidectomy specimen revealed the effacement of thyroid architecture by abnormal and extensive lymphoid follicles. Immunohistochemistry revealed CD20, CD10, BCL2, and BCL6 positivity in the lymphoid follicles. FDG-PT CT scan demonstrated no evidence of lymphoma elsewhere. So, a e final diagnosis of follicular lymphoma of the thyroid was made.

**Conclusion:**

Due to the rarity and low prevalence of primary follicular lymphoma of the thyroid and challenging in its differentiation from Hashimoto's thyroiditis with dense lymphoplasmacytic infiltration and formation of lymphoid follicles, histopathologic diagnosis should be confirmed by immunohistochemical studies.

## Introduction

Primary lymphoma of the thyroid gland accounts for 2-5% of extranodal non-Hodgkin lymphomas (1). Most primary thyroid lymphomas are non-Hodgkin type and have a B-cell phenotype (2). The most frequent primary thyroid lymphomas are MALT lymphomas and diffuse large B cell lymphomas (1). Only 3-5% of total cases are represented by follicular lymphoma, and it occurs approximately three times more frequently in women than in men and typically affects those over 50 years of age (median age, 60-65 years) (1). Most lymphomas in the thyroid gland arise from chronic lymphocytic thyroiditis. Histological features of primary thyroid lymphoma, including rare subtypes, are the same as those of malignant lymphoma arising from sites other than the thyroid (2). We report this case because of its rarity.

## Case Presentation

A 62-year-old female who was a known case of dyslipidemia and hypertension, presented with an enlarged, painless swelling in front of the neck, which showed a gradual increase in size over the past month. There was no history of dyspnea, dysphagia, hoarseness of voice, or B symptoms (weight loss, fevers, and night sweats). Physical examination showed a non-tender, firm swelling measuring 1.5 cm × 1.5 cm on the right side of the neck. The swelling was moving with deglutition. No cervical lymphadenopathy was noted. Other systems were within normal limits. Thyroid function tests were found to be normal. 

Ultrasound examination of the thyroid showed the right lobe measured 1.8 cm× 1.5 cm x 5.4 cm with two well-defined, wider than taller, predominantly cystic nodules of 1.2 cm × 2.2 cm and 1.5 cm × 2.3 cm, showing echogenic debris and peripheral vascularity. The left lobe was normal. There was no significant cervical lymphadenopathy. A diagnosis of thyroiditis was suggested with two degenerated adenomatoid nodules in the right lobe without retrosternal extension (TIRADS-2).

**Fig. 1:**
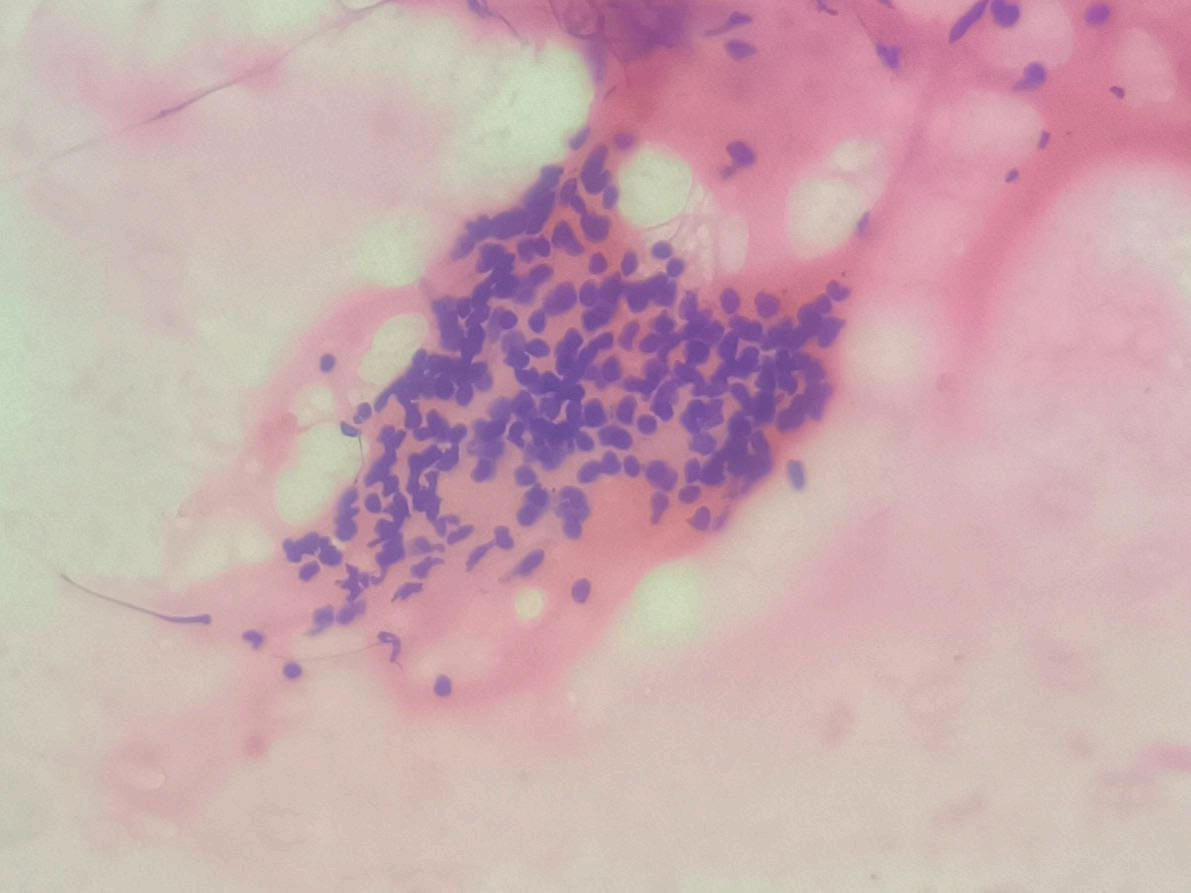
Fine needle aspiration cytology of the thyroid showing lymphocytes admixed with thyroid follicular cells, 400x (A), H & E stain

Fine needle aspiration cytology showed a cellular smear with follicular cells arranged in clusters and scattered singly. The cells exhibited mild anisonucleosis and moderate eosinophilic cytoplasm and round nuclei. The background showed lymphocytes and colloids mixed with blood. The diagnosis given was nodular colloid goiter with lymphocytic thyroiditis (Bethesda category II) (Fig. 1 & Fig. 4).

**Fig. 2:**
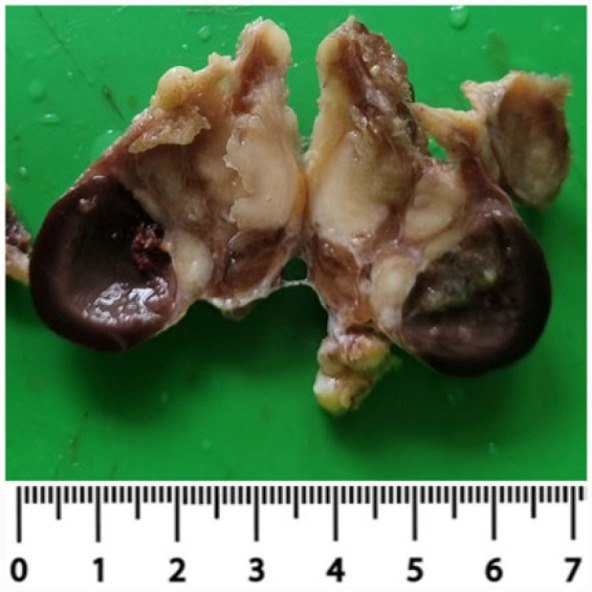
Gross image of the right hemithyroidectomy specimen (cut surface)

The patient underwent the right hemithyroidectomy. Gross examination of the right hemithyroidectomy specimen weighed 15gm, measuring 5cm x 4cm x 3cm. The surface appeared nodular and congested. Two perithyroidal lymph nodes measuring 1 cm × 1 cm x 1cm were also seen. The cut section of the thyroid showed an irregular, large, grey-white area involving the upper pole of the right lobe measuring 4cm × 2cm × 2cm. The rest of the thyroid showed multiple small colloid-filled nodules, which showed degenerative changes (Fig. 2). 

**Fig. 3:**
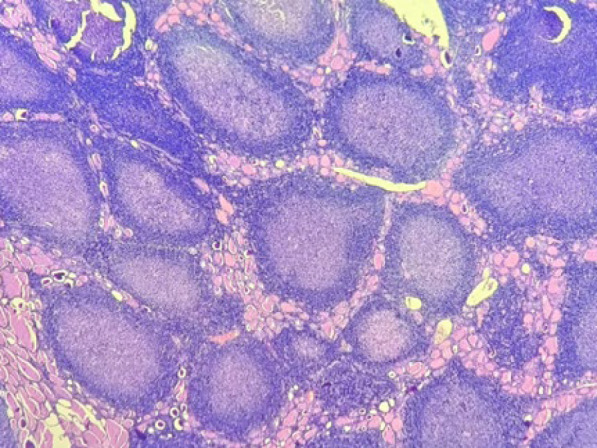
The histopathologic image showing neoplastic lymphoid follicles infiltrating the thyroid tissue, 100x, H & E stain

On microscopy, the sections from the grey-white area of the right lobe of the thyroid showed effaced thyroid architecture by extensive and dense lymphoid infiltrate composed of the closely packed abnormal lymphoid follicles (Fig.3). The follicles showed lack of polarization with attenuated mantle zone and absence of tangible body macrophages (Fig.5). The cells were predominantly centrocytes with cleaved nuclei, admixed with centroblasts. The lymphoid cells were seen infiltrating the perithyroidal adipose tissue (Fig.6). The background showed features of Hashimoto’s thyroiditis. Adjacent small lymph nodes also showed effaced architecture with infiltration by similar cells. Further immunohistochemistry showed CD20, CD10, BCL2, and BCL6 positivity in the tumoral cells. CD23 was positive in the follicular dendritic cells. CyclinD1 was negative. The ki67 proliferation index was 10% (Fig.7). The diagnosis given was follicular lymphoma of the thyroid (grade 2).

**Fig. 4:**
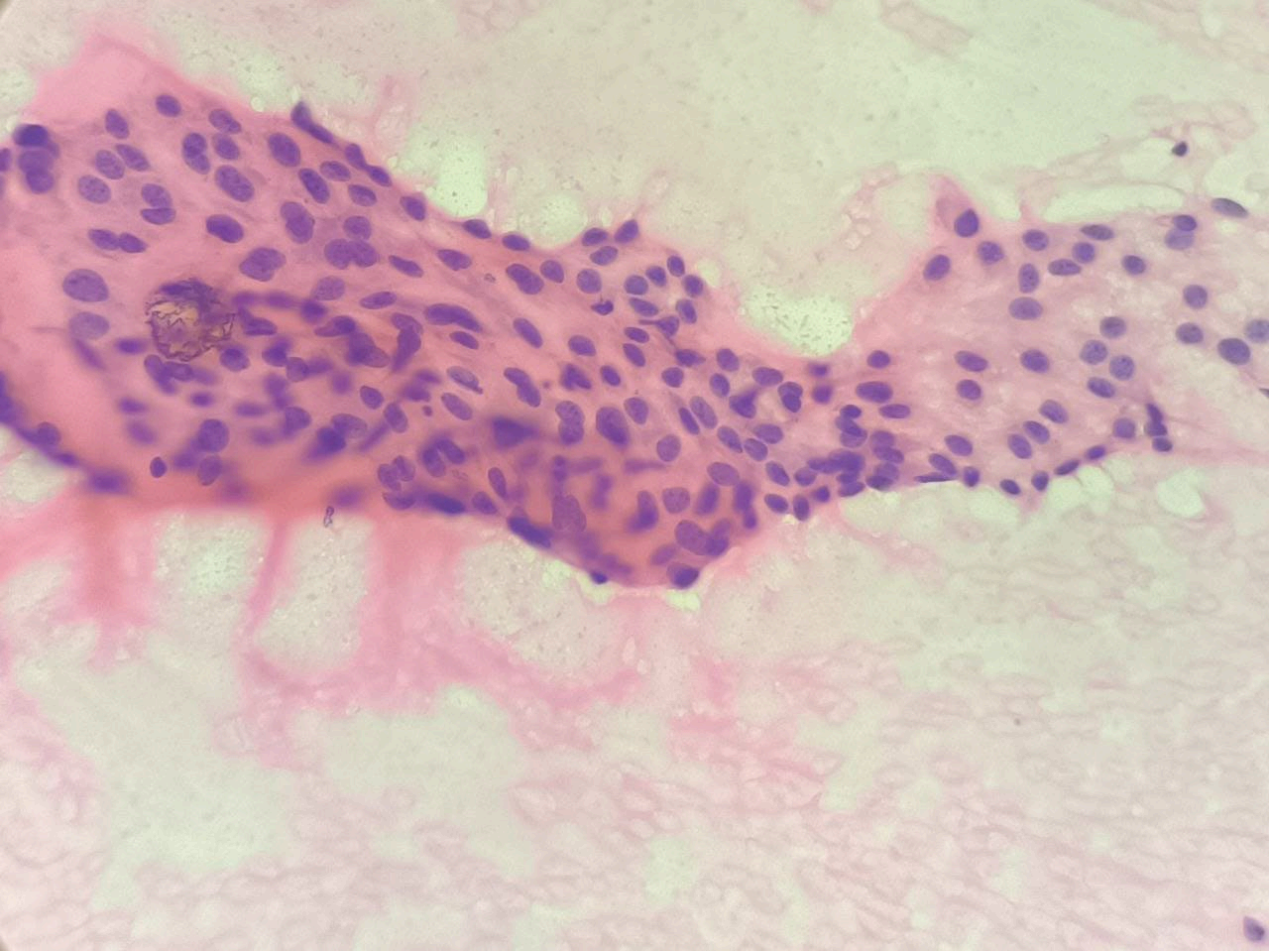
Fine needle aspiration cytology of the thyroid showing lymphocytes admixed with thyroid follicular cells, 400x (B), H & E stain

A whole-body FDG-PET scan was performed. There was no focal abnormal tracer uptake in the left lobe, or any other metabolic activity seen elsewhere in the visualized body survey. So, a final diagnosis of primary follicular lymphoma of the thyroid was made. The patient was treated with 15 fractions of external beam radiation therapy and was doing well on the 6th-month follow-up.

**Fig. 5:**
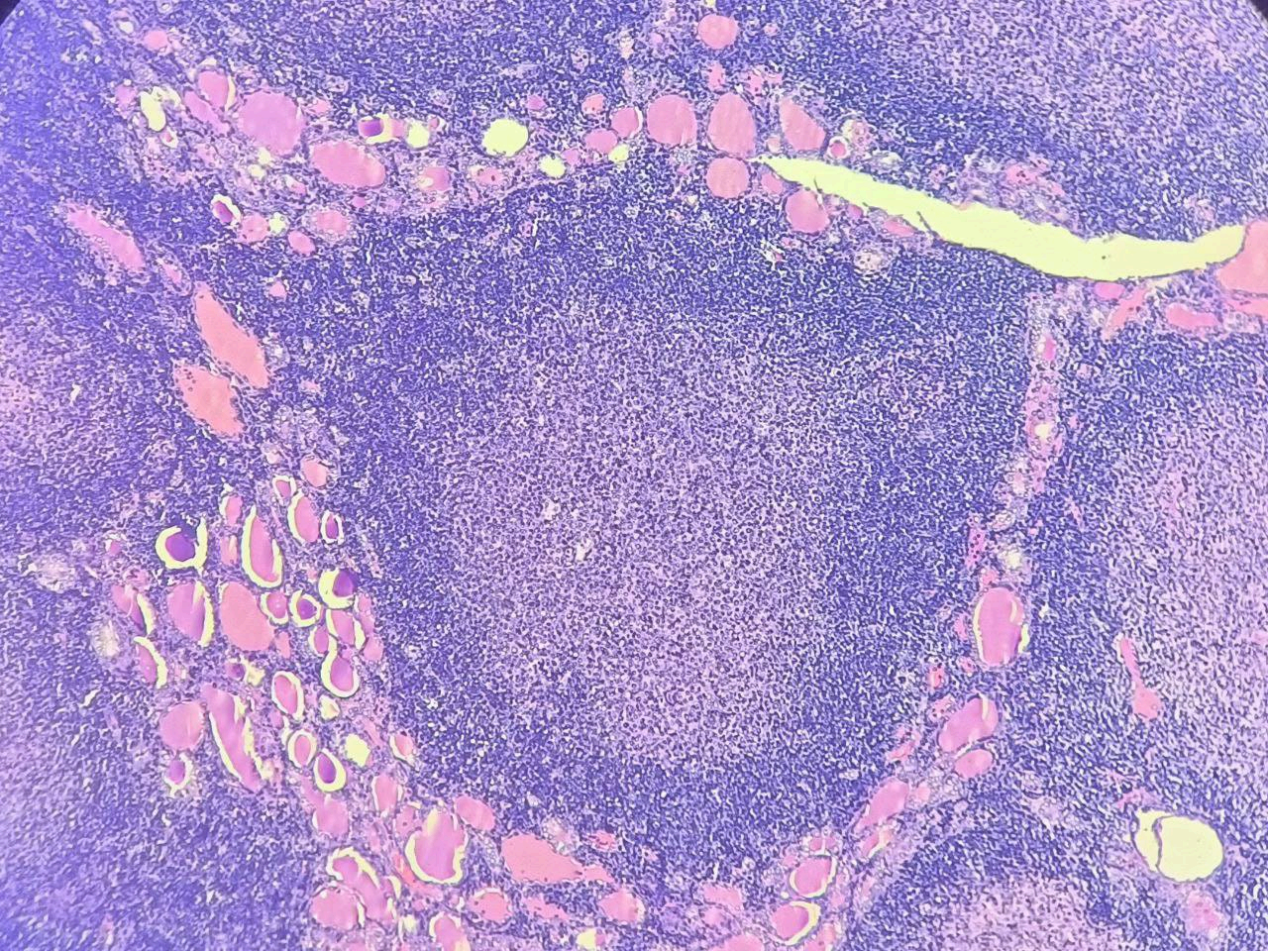
The Histopathologic image showing neoplastic lymphoid follicle infiltrating the thyroid tissue, 400 x, H&E stain

**Fig. 6:**
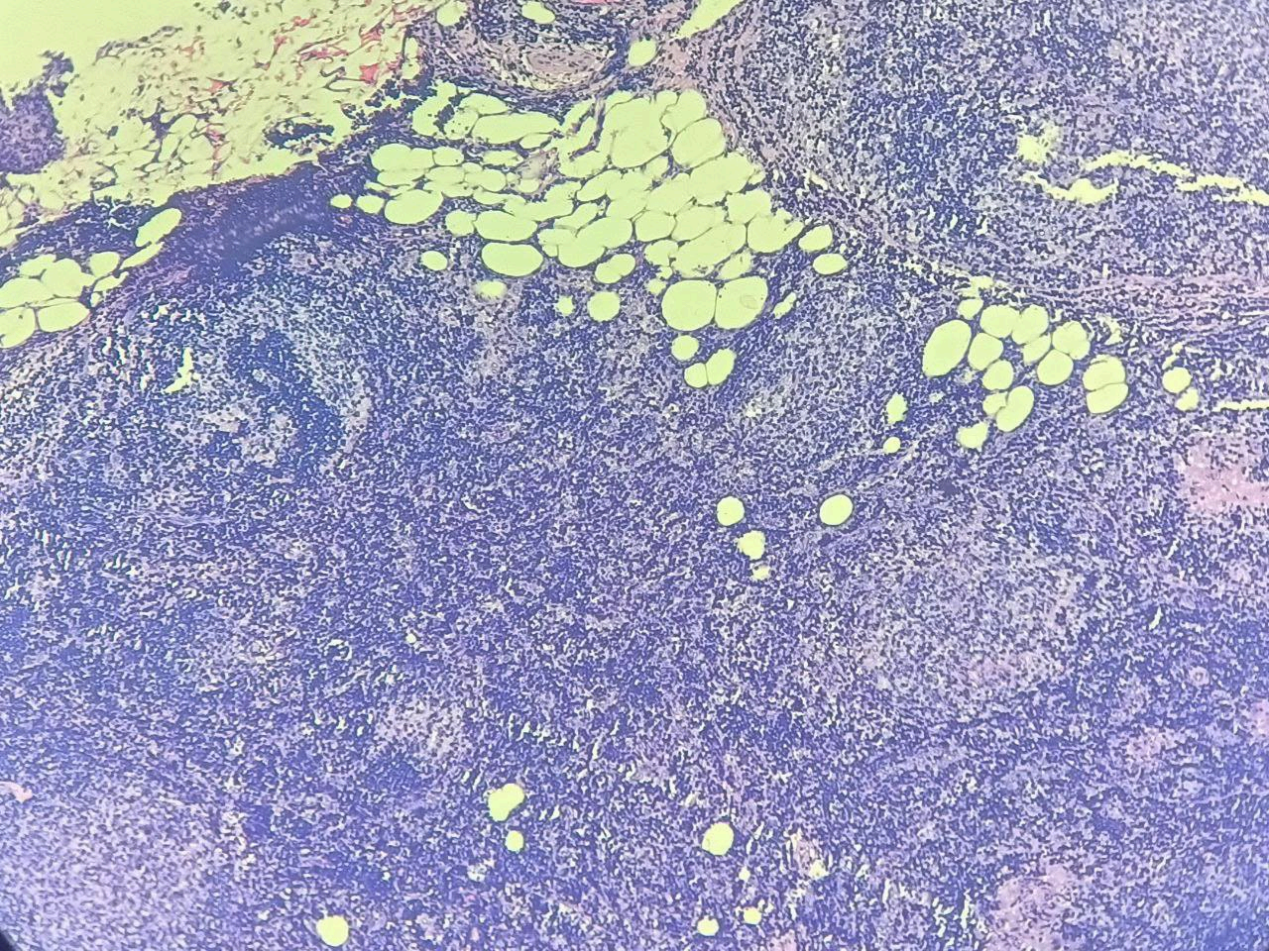
The histopathologic image showing lymphoid cells infiltrating the perithyroidal adipose tissue, 100x, H&E stain

**Fig. 7:**
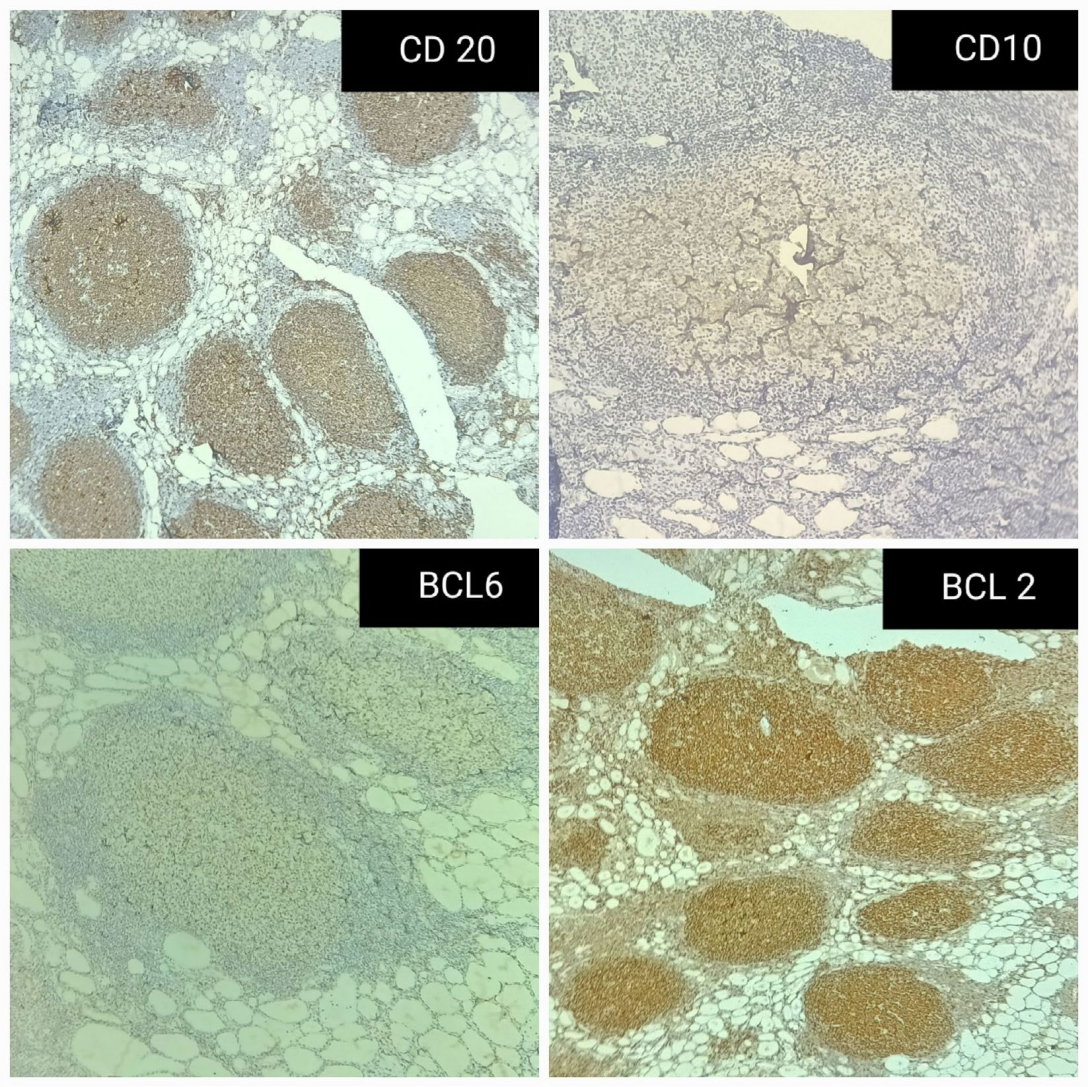
The immunohistochemical images show CD20, CD10, BCL2, and BCL6 positivity in the lymphoid follicles.

## Discussion

Normal thyroid gland does not have any components of lymphoid tissue. In cases where intrathyroidal lymphoid tissue is seen, it is usually associated with autoimmune diseases like Hashimoto’s thyroiditis (HT) (3). Interestingly, although the incidence of HT in patients with primary thyroid lymphoma (PTL) approaches 80%, only 0.6% of the patients with HT will go on to develop PTL (4). Histologically, this acquired lymphoid tissue closely resembles mucosa-associated lymphoid tissue (MALT) and can evolve into lymphoma (3). The association of HT and lymphoma is postulated to result from developing intrathyroidal lymphoid tissue in HT. It has also been suggested that for the evolution of PTL, antigenic stimulation that is distinct to the thyroid microenvironment is required. Further strengthening this hypothesis is that greater than 50% of thyroid lymphoma subjects have a history or co-existing diagnosis of chronic lymphocytic thyroiditis, positing the part of antigenic stimulation in the pathogenesis (4).

Compared with the general population, the relative risk of a patient with lymphocytic thyroiditis developing lymphoma is 40 to 80 times. On average, it takes 20 to 30 years to develop lymphoma after the onset of lymphocytic thyroiditis (5). In reported cases of follicular lymphoma, the ratio of females to males is 4.5:1, while ages range from 26 to 72 years (median, 50 years) (2).

In a study conducted by Derringer *et al.*, out of 108 studied cases of primary thyroid lymphomas, clinically, all the patients presented with a mass in the thyroid gland that was noticeably enlarging in 72% of the cases. Symptoms resulting from compression/infiltration of the neck organs, such as dyspnea, dysphagia, choking and/or coughing, and hemoptysis, were reported in 31% of the cases (n 33). Few patients experienced pain. Overall, the duration of the symptoms ranged from few days to 36 months. Cervical/perithyroidal lymph nodes were the most common nodal sites, usually identified at or shortly after the initial surgery, followed by mediastinal and abdominal lymph nodes. Extranodal involvement was found in 8.3% of all the cases. Of the 108 cases, 30 were MZBL, 36 were mixed DLBCL and MZBL, 41 were DLBCL without MZBL, and one FCL. All lymphomas were composed of B-lymphocytes that effaced thyroid parenchyma. Infiltration of the perithyroidal skeletal muscle and/or adipose tissue by the lymphoma cells was identified in 70% of the cases (75 of 107 cases assessable), inclusive of the FCL case. Overall, 70 of 108 (65%) patients were alive at the last follow-up, which translates into a raw survival of 60% at 5 years (5). Majority of the patients (30–60%) were biochemically euthyroid at presentation. Later, many patients developed hypothyroidism and had thyroid hormone replacement therapy because of the frequent coexistence of HT. Circulating antibodies to thyroid peroxidase were observed in 60% of the patients. Fever, night sweats, and weight loss, which are the classic B-type symptoms, were seen less commonly (4).

MALT lymphomas of the thyroid tend to present localized disease and usually respond favorably to therapy with complete remission in almost all the cases and only rare relapses. In contrast, follicular lymphoma is a neoplasm of germinal center B cells, which typically present with nodal or disseminated disease. Most patients experience a protracted clinical course characterized by transient responses to therapy and multiple relapses, often ending in death from resistant disease or transformation to DLBCL ( 1 ).

In a retrospective analysis of 26 patients with thyroid lymphoma (TL), they were mostly females, with a median age of 59 years , presenting as rapidly growing nodular goiter with or without cervical adenopathy, without symptoms related to lymphoma for 81% and hypothyroidism for 61%. A previous history of Hashimoto’s thyroiditis was observed in 11 patients. The following subtypes of lymphoma were noticed: 50% had diffuse large B cell lymphoma, 23% had mucosa-associated lymphoid tissue (MALT) lymphoma, 12% had follicular lymphoma, 7% had Hodgkin’s disease, 4% had small lymphocyte and 4% had Burkitt’s lymphoma (3).

FNAC does a primary pathological examination of the thyroid lesion. It is a challenge for the pathologist to differentiate lymphoma and chronic thyroiditis, particularly when the inflammatory exudates breach the normal thyroid architecture. Low-grade lymphomas can be mistaken for chronic thyroiditis. Also, thyroiditis leads to cellular aggregates known as lymphoepithelial lesions and prominent germinal centers. Chronic lymphocytic thyroiditis is characterized by a combination of T and B lymphocytes, predominantly T cells. Scattered larger lymphoblasts, plasma cells, and immunoblasts are also observed. Core needle biopsy or surgical biopsy is considered the gold standard for diagnosing and assessing the aggressiveness of the tumor. The advantage of biopsy over FNAC is that there is more tissue yield with maintained architecture, which allows for differentiating among HT, PTL, and anaplastic carcinoma, which is arduous to achieve with FNAC (4).

Immunocytochemical staining may be helpful when lymphoma is suspected of demonstrating both lineage and clonality of the cell population. High-grade thyroid lymphomas are easily diagnosed based on cytomorphological characteristics, and immunocyt-ochemistry can confirm suspicious cases. A diagnosis of low-grade thyroid lymphoma is more challenging, and distinguishing features include abundance of lymphoid tissue and a high proportion of intermediate centrocyte-like cells. ICC can confirm suspicious cases. False-negative results seem to be caused by inadequate sampling (6).

Histological features of thyroid lymphoma include effacement of the thyroid architecture by extensive and dense lymphoid infiltrate composed of numerous lymphoid follicles amongst a variably prominent interfollicular density of diffuse components. The lymphoid follicles show absence of polarization, attenuation of mantle zones, and lack of tangible body macrophages. The germinal center contains characteristic centrocytes and centroblasts in variable proportions (2). Histologically, follicular lymphomas usually grow with a predominant follicular pattern and a variable but smaller interfollicular component (1). An important diagnostic finding is the packing of follicular lumina by lymphoid cells (“lymphoepithelial lesions”), a feature usually not present in thyroiditis (7). 

Immunohistochemical and genetic features recognize two distinct groups of thyroid follicular lymphoma. Group I is similar to the classic nodal follicular lymphoma and carries t(14;18)/ IGH-BCL2. The patients usually have high-stage disease (II-IV) and achieve partial or complete remission. Most cases show histologic grade I or II, a CD10+, BCL-2+ immunophenotype, and BCL2 gene rearrangement. Group II is similar to the minority BCL2− follicular lymphoma of extranodal sites or lymph nodes and lacks IGH-BCL2. The patients often have early-stage disease (stage I) and usually attain complete remission after treatment. Most cases show histologic grade III, variable immunostaining for CD10, negative immunostaining for BCL-2, and lack of BCL2 gene rearrangement (1,8). 

Surgery alone has been proposed for the management of localized intrathyroidal follicular lymphoma. Control of distant metastasis of the disease is achieved by chemotherapy, while local control of lymphoma is attained by radiation therapy (4).

In a retrospective analysis done by Thieblemont *et al.* complete remission in response to treatment was achieved in 19 of the 25 (76%) patients. One case each of DLCL, Hodgkin’s disease, and follicular lymphoma relapsed at 6 months, 22 years, and 10 years, respectively. Patients who underwent surgical management for MALT lymphoma did not relapse after achieving remission. Progression of the disease occurred in all of these patients in a median time of 5 months (range, 1–20.5 months) (3).

Study done by Graff-Baker et al. of 1408 patients with PTL over 32 years of follow-up from the Surveillance, Epidemiology, and End Results Database of the National Cancer Institute, the median overall survival for all cases was 9.3 years, 5-year overall survival was 66%, and the disease-specific survival was 79%. Older age, advanced stage, histologic subtype, and lack of radiation/surgical treatment are associated with worse survival. Thyroid resection offers benefits only for patients with stage I disease. Management of PTL requires multidisciplinary collaboration (9).

In a follow-up done by Bacon *et al. *in a study on 15 patients with follicular thyroid lymphoma, after a median follow-up of 44 months (range, 10 204 months), 11 patients could survive with no evidence of lymphoma. Ten of these achieved a complete remission following initial therapy and did not relapse; the other relapsed with DLBCL following an initial partial response but achieved a complete response to salvage therapy. Four patients, three of whom achieved only a partial response, had died of progressive or relapsed transformed disease. One patient had a diagnostic needle core biopsy followed only by observation and remained alive with lymphoma four years from diagnosis (1). 
